# Prevalence and Associated Family Factors of Sibling Bullying Among Chinese Children and Adolescents

**DOI:** 10.3389/fpsyg.2022.892598

**Published:** 2022-07-14

**Authors:** Zaihua Qing, Yankun Ma, Xiaoqun Liu

**Affiliations:** ^1^Hunan University of Finance and Economics, Changsha, China; ^2^School of Education, Teachers College, Guangzhou University, Guangzhou, China; ^3^Xiangya School of Public Health, Central South University, Changsha, China

**Keywords:** sibling bullying, family environment, Chinese, children and adolescents, family factors

## Abstract

Sibling bullying is the most common form of aggression within family worldwide, while the prevalence and correlations of sibling bullying is little known in China. The current research focused on the association between family factors and sibling bullying among Chinese adolescents, and explore sex differences in sibling bullying in the context of Chinese culture. A cross-sectional study was conducted to explore the characteristics of sibling bullying by sampling 6302 children and adolescents who had at least 1 sibling living in the household. Of the participants, 1827 (29.0%) were involved in sibling bullying over the past half year, and pure victims, pure bullies, and bully-victims were 486 (7.7%), 510 (8.1%), and 831 (13.2%), respectively. Family factors of sibling bullying were partly different between boys and girls. Parental absence of both father and mother was a risk factor of being a pure bully and a bully-victim for boys, and of being a pure victim for girls. Parental son preference increased the odds of being a pure victim and a bully-victim for boys, and of being all roles of sibling bullying involvement for girls. Besides, parent–parent violence, parent–child violence, and living with a single parent were risk factors of sibling bullying. The results underline the importance of home environment on sibling relationship, and intervention of sibling bullying should include improving family climate.

## Introduction

Sibling bullying refers to any unwanted aggressive behavior by a sibling, which is featured by an observed or perceived power imbalance and repetitiveness (Wolke and Samara, 2010; Wolke et al., 2015). Sibling bullying is a common incident in childhood and adolescence within households worldwide (Khan and Cooke, 2013; Yu et al., 2017). According to a systematic study, nearly 50% of children are involved in sibling bullying every month, and 16–20% experience sibling bullying several times a week (Menesini et al., 2011; Wolke and Skew, 2012). Though sibling bullying is usually considered by parents or researchers as a normal or harmless phenomenon (Kettrey and Emery, 2006; Dale et al., 2014), there is ample evidence to support that sibling bullying can predict a number of internalizing and externalizing problems in childhood or early adulthood, which include depression, anxiety, self-harm behavior, and even suicide (Bowes et al., 2014; Jasmin and Anat, 2018; Foody et al., 2020). Sibling bullying was associated with clinical diagnosis of depression and suicidal ideation as well as suicidal self-harm (Dantchev et al., 2019; Sharpe et al., 2021).

However, compared with the volume of studies conducted in the western countries (Dantchev and Wolke, 2018; Dantchev et al., 2018), there are a paucity of studies with regard to aggressive behavior between siblings in the East (Yu et al., 2017), especially in China. Few is known about the characteristics of sibling bullying in this one of the most populous countries. With the one-child policy have been replaced by the two-child policy since 2011, there is an increasing number of families with two or multiple children in both rural and urban areas of China (Zeng and Hesketh, 2016). Therefore, it is necessary to understand the prevalence or factors relating to sibling bullying among Chinese population.

Family climate is the primary environment in where children interact with their sibling(s) (Wolke et al., 2015). According to family systems theory, family members are interdependent and can affect one another in a mutual and continuous way (Feinberg et al., 2012). Moreover, from a perspective of social learning theory, behavior learning of children can occur through direct experience or indirect observation of others’ behavior (Goodman et al., 2015). Several existing studies have identified significant associations between sibling bullying and family environmental factors, which were generally categorized into three categories: family structure, socioeconomic factors, and parental behavior (Wolke et al., 2015). Family structural factors generally include family composition (Tucker et al., 2014a), number of siblings (Tippett and Wolke, 2015), sibling’s gender (Menesini et al., 2011), age difference between siblings (Tucker et al., 2013), and birth order (Zeng and Hesketh, 2016). The resource control theory suggests that social group (for example, family systems) asymmetry may foster competitive behavior for social resources (Hawley, 1999), Siblings who are very asymmetrical in family structure, such as age, size, sex, birth order, abilities and so on, may provoke sibling conflict or sibling bullying in order to access to limited parental resources (e.g., affection, attention, and material goods) (Toseeb et al., 2020). For example, when a new sibling was born in the family, the first-born child may face the risk of losing some parental resources and then perpetrate sibling bullying in order to regain parental resources (Toseeb, 2021). Socioeconomic factors include family income (Eriksen and Jensen, 2009), parental education (Dantchev et al., 2018), and other characteristics that can represent socioeconomic status of household (Wolke et al., 2015). Research has shown that low-economic status was associated with sibling victimization, and economic hardship and a lack of financial resources were associated with greater physical aggression between siblings (Hardy, 2001). High socio-economic parents usually require more cooperative behavior among siblings, and they are particularly sensitive to sibling bullying and more likely to inhibit it effectively (Tucker et al., 2013). Parental behavior mainly include parental violence and parent–child violence (Button and Gealt, 2010; Radford et al., 2013). Studies have suggested that children may imitate the behavior patterns (e.g., aggression behavior) of their family members (Bandura, 1978), and children who witness or experience violence against parent or sibling (parent assault of a sibling) were at greater risk of becoming victims of sibling bully (Renner et al., 2020).

Apart from these family characteristics, there are other factors within household may also have effect on sibling bullying, such as parental absence and son preference, which root from the social and cultural background of the oriental countries. In China, there are over 61 million left-behind children, who have being left behind in home by one or both parents who migrate for economic reasons (Zhao et al., 2015; Chen and Ling, 2016). Previous studies had indicated that parental absence has strong links to psychological and behavioral problems of children (Zhao et al., 2014; Guo et al., 2015). Another potential factor that differs from the traditional context of the West is son preference. Chinese couples, particularly those in rural areas, still have historically a strong son preference, which refer to parents value sons more than daughters (Murphy et al., 2011). The different position of boys and girls within the family is reflected in such a Chinese traditional saying, “a son keeps incense at the ancestral alter burning” while “raising a daughter is like pouring water to other people’s field” (Shi, 2009). Therefore, son preference generally plays a sizeable impact on parents’ treatment of their offspring and often results in discriminatory behavior toward girls, which may motivate aggressive behavior between sons and daughters (Hesketh et al., 2011; Volk et al., 2012).

Current studies have shown that sex differences in sibling bullying with mixed results. Researchers have found that boys reported higher acceptability of sibling bullying as well as more likely to be involved in perpetrating sibling bullying than girls (Zhao et al., 2014), and girls have a higher rate being victims of sibling bullying (Button and Gealt, 2010). Some studies have also found no sex differences in sibling bullying (Wolke and Samara, 2010). Further work is needed to better understand this issue.

To the best of our knowledge, there is little research has assessed the association between sibling bullying and home environment based on Chinese population. What’s more, there are several family characteristics that may be associated with sibling bullying have not been explored, including parental absence and son preference. Therefore, the current research focused on the association between family factors and sibling bullying by a cross-sectional approach among Chinese adolescents, and explore sex differences in sibling bullying in the context of Chinese culture.

## Materials and Methods

### Procedure and Participants

This cross-sectional study was conducted from April to July, 2019. The participants were recruited from Hunan Province, China by a multi-stage cluster sampling. We used a geography-based stratified sampling frame which included three cities selected randomly from southern, central, and northern parts of the province, respectively. Three junior high schools and three senior high schools were selected randomly from each chosen city. Within each school, all the students of grade 7–12 were invited to the research.

The study received the approval from the Ethical Committee of Xiangya School of Public Health, Central South University. Before the investigation in each school, a survey team was established, which included several teachers and two investigators. All members of these teams received training for research tool, study process, and quality control. Informed consent was obtained from the principal of each chosen school, the student who participated in the study, and their parents. In addition, the purpose of the study as well as the questionnaire sections were explained to all respondents by investigators. They were free to discontinue their participation at any time, and they were assured of the anonymity and confidentiality of the answers that they provided. The self-reported questionnaire averaged 30 min in length.

We sent out 8918 questionnaires to the students from 18 sampled schools, and recollected 8717 questionnaires without missing values, with a response rate of 97.7%. The current study focused on 6302 children and adolescents aged 9–18 who had at least 1 brother or sister in their family.

### Measurements

#### Sibling Bullying

Sibling bullying was measured by using the Chinese version of Olweus Bully/Victim Questionnaire (OBVQ). First, sibling bulling victimization was assessed by asking that have you ever been bullied by siblings in the last 6 months using the following six items: (1) having been hit, kicked, pushed, or shoved; (2) having belongings been taken or damaged; (3) having been called nasty name; (4) having been made fun of; (5) having been kept out of things on purpose, excluded from the group or completely ignored; and (6) they told lies or spread rumor about you and/or tried to make others dislike you. Second, sibling bullying perpetration was measured by asking that have you ever bullied siblings over the past half a year using the six items as above. The frequency was coded on a 5-point scale ranging from 1 to 5 (1 = never happened, 2 = only once or twice, 3 = two or three times a month, 4 = about once a week, 5 = several times a week). Respondents were considered to be involved in sibling bullying victimization or perpetration if they chose 3, 4, or 5 for any items from OBVQ (Sharpe et al., 2021). The Chinese version of OBVQ showed good reliability according to the existing literature (Zhang and Wu, 1999). In this study, the internal consistency reliability (Cronbach’ alpha coefficient) for victimization and perpetration of sibling bullying were 0.79 and 0.86, respectively.

For roles of sibling bullying involvement, a pure victim was defined as he/she was involved in victimization but not engaged in perpetration, a pure bully was classified as he/she perpetrated bullying behavior but not been bullied, a bully-victim was defined as he/she experienced both victimization and perpetration of bullying. Those who neither bullied siblings nor were bullied by siblings were classified as “non-involved” (Toseeb et al., 2018).

#### Potential Family Factors

In the current study, the family factors were classified into three aspects: structural factors, socioeconomic factors, and parental behavioral factors.

Structural factors included six variables: family composition, parental absence, number of siblings (1, 2, 3, or more), birth order (first, second, third, or other), sibling’ gender, and age difference between siblings (1 = 0–4 years, 2 = over 4 years). Family composition was categorized into three groups: children living with (1) two parents, (2) single parent, and (3) other caregivers. According to the definition of left-behind children (Wang et al., 2014), parental absence was obtained by the question “Who had gone to a city for a job over the past 6 months in your family?” The answer had four options (1) none, (2) father, (3) mother, and (4) both. Socioeconomic factors included three variables: family location, parental education, and perceived family income. Family location had two options (urban, rural). Parental education for the parent with the most education, representing (1) primary school or less, (2) junior high school, (3) senior high school, and (4) college or more. The perceived family income was assessed by the question “How would you like to evaluate your family income within your region?” The answer was measured by 3 Likert scales ranging from 1 to 3 (1 = poor, 2 = medium, 3 = good). Parental behavioral factors included three variables: parental violence, parent–child violence, and son preference. Parental violence was measured by the question “How often your parents or other caregivers fight with each other in the last 6 months?” Parent–child violence was measured by the question “How often your parents or other caregivers hit or abused you in the last 6 months?” The frequency of two questions as above coded on a 5-point scale, ranging from 1 to 5 (1 = never happened, 2 = only once or twice, 3 = two or three times a month, 4 = about once a week, 5 = several times a week). If the respondent answered 3, 4, or 5, parental violence or parent–child violence was coded as 1, otherwise, it was coded 0. Son preference was measured by the question “How much do you think your parents or caregivers prefer sons to daughters?” The answer was measured by 5 Likert scales ranging from 1 to 5 (1 = not at all, 2 = a little bit, 3 = moderately, 4 = quite a bit, 5 = extremely). If the respondent answered 3, 4, or 5, Son preference was coded as 1, otherwise, it was coded 0.

Demographic factors of participants included gender, age, and grade (7–12).

### Data Analysis

The characteristics of participants, prevalence of sibling bullying, and percentage of roles of sibling bullying involvement were summarized by descriptive statistics [*n* (%)]. Chi-square test was used to analyze difference in prevalence of sibling bullying as well as in percentage of roles of sibling bullying involvement between boys and girls. Multinomial logistic regression analysis was employed to explore the potential family factors of sibling bullying. For assessing underlying gender differences in son preference, two models were conducted for boys and girls separately after controlling demographic factors. Dependent variables of the two models were the roles of sibling bullying involvement, which included four categories: (0) non-involved group, (1) pure victim group, (2) pure bully group, and (3) bully-victim group. The associations between potential family factors and sibling bullying were reported by odd ratios and 95% confidence intervals [OR (95% CIs)]. The significance level was set at *p* < 0.05. All of the data analysis was conducted by SPSS 22.0.

## Results

### The Characteristics of the Sample

Of 6302 children and adolescents in the study, 45.2% were boys and 54.8% were girls. The participants aged 9–12 (56.9%) were more than those aged 13–18 (43.1%), and the average age was 11.69 (SD 1.28).

In terms of family structure, most of children lived in families with two parents (90.1%). About 40% of the sample were identified as left-behind children, more specifically, 15.6% with father left, 4.4% with mother left, and 20.5% with parental absence of both father and mother. Up to one third of participants had only one brother or sister (75.8%). For family socioeconomic status, the sample was approximately evenly divided by family location. The parental education distribution was primary school or less (17.4%), junior high school (55.7%), senior high school (21.7%), and college or more (5.1%). Most of children believed that their family income ranked medium within their region (78.3%). In parental behavior, 11.8% of participants had witnessed domestic violence between parents and 4.3% had experienced parent–child violence over the past half year. Nearly 30% of children believed that their parents or caregivers had son preference (Table 1).

**TABLE 1:** The characteristics of the sample.

Factors	*n*	%
**Gender**		
Boy	2846	45.2
Girl	3456	54.8
**Age**		
9–12	3585	56.9
13–18	2717	43.1
**Family composition**		
Two parents	5676	90.1
Single parent	265	4.2
Adopted or other	361	5.7
**Parental absence**		
Non	3749	59.5
Father	986	15.6
Mother	277	4.4
Both	1290	20.5
**Sibling’s gender**		
Boy	3184	50.5
Girl	3118	49.5
**Age difference**		
0–4	2116	33.6
5 or more	4186	66.4
**Number of siblings**		
1	4779	75.8
2	1201	19.1
3 or more	322	5.1
**Birth order**		
First	2978	47.3
Second	2833	45.0
Third or other	491	7.8
**Family location**		
Urban	2985	47.4
Rural	3317	52.6
**Parental education**		
Primary school or less	1098	17.4
Junior high school	3513	55.7
Senior high school	1369	21.7
College or more	322	5.1
**Perceived family income**		
Poor	930	14.8
Medium	4935	78.3
Good	437	6.9
**Parental violence**		
No	5559	88.2
Yes	743	11.8
**Parent–child violence**		
No	6030	95.7
Yes	272	4.3
**Son preference**		
No	4460	70.8
Yes	1842	29.2
Total	6302	100.0

### Prevalence and Percent of Roles of Sibling Bullying Involvement

Within the sample, 1827 (29.0%) involved in sibling bullying in the last half a year. Specifically, 1317 (20.9%) were bullied by their siblings, while 1314 (21.3%) perpetrated bullying behavior toward their siblings. Boys (22.2%) were higher than girls (19.8%) in prevalence of sibling bullying victimization.

With respect to roles of sibling bullying involvement, 486 (7.7%) of children reported being victims only, 510 (8.1%) reported being pure perpetrators, and 831 (13.2%) reported being both victims and bullies. Significant gender difference was only found among pure victims, and boys (8.5%) were higher than girls (7.1%) in percentage of pure victims (Table 2).

**TABLE 2:** Prevalence and percent of roles of sibling bullying by gender.

	Total (*n* = 6302)		Boy (*n* = 2846)		Girl (*n* = 3456)		*P*-value
	*n*	%	*n*	%	*n*	%	
**Prevalence**							
Victimization	1317	20.9	631	22.2	686	19.8	0.024
Perpetration	1341	21.3	620	21.8	721	20.9	0.373
**Roles of involvement**							
Non-involved	4475	71.0	1985	69.7	2490	72.0	0.108
Pure victim	486	7.7	241	8.5	245	7.1	0.041
Pure bully	510	8.1	230	8.1	280	8.1	0.977
Bully-victim	831	13.2	390	13.7	441	12.8	0.271

### Family Factors Associated With Sibling Bullying

For boys, those living with a single parent were at greater risk of being a bullying (OR = 2.07, 95% CI 1.12–3.82) than those living with two parents. Being left behind by both father and mother was at greater risk of being a bully (OR = 1.69, 95% CI 1.18–2.42) and being a bully-victim (OR = 1.35, 95% CI 1.00–1.81). The male participants whose sibling was a brother had higher odds of being all three roles of sibling bullying involvement. Age difference between siblings was a protective factor for the three roles of sibling bullying involvement. Children born third or more were at greater risk of being a victim than those first-born (OR = 1.99, 95% CI 1.21–3.27). Children from medium-income families were at less risk of being a victims than those from poor-income families (OR = 0.64, 95% CI 0.45–0.89). Both parental violence and parent–child violence were risk factors of sibling bullying involvement. Those boys whose parents had son preference were more likely to be a victim (OR = 1.90, 95% CI 1.41–2.58) and a bully-victim (OR = 2.29, 95% CI 1.79–2.92) compared with those who were not involved in sibling bullying (Table 3).

**TABLE 3:** Multinomial logistic regression of roles of sibling bullying among boys (*N* = 2846).

Factors	Pure victim	Pure bully	Bully-victim
**Family composition**			
Two parents	Ref	Ref	Ref
Single parent	0.52 (0.23, 1.15)	2.07 (1.12, 3.82)*	1.04 (0.58, 1.87)
Adopted or other	0.69 (0.31, 1.54)	1.02 (0.56, 1.86)	1.33 (0.82, 2.16)
**Parental absence**			
No	Ref	Ref	Ref
Father	0.87 (0.58, 1.31)	1.28 (0.86, 1.90)	1.23 (0.89, 1.69)
Mother	1.08 (0.55, 2.12)	1.82 (0.95, 3.48)	1.44 (0.83, 2.49)
Both	1.06 (0.74, 1.52)	1.69 (1.18, 2.42)**	1.35 (1.00, 1.81)*
**Sibling’s gender**			
Girl	Ref	Ref	Ref
Boy	1.36 (1.03, 1.81)*	1.37 (1.03, 1.83)*	1.79 (1.41, 2.28)***
**Age difference**			
0–4	Ref	Ref	Ref
5 or more	0.66 (0.49, 0.88)**	0.62 (0.46, 0.84)**	0.39 (0.31, 0.49)***
**Number of siblings**			
1	Ref	Ref	Ref
2	1.01 (0.68, 1.49)	1.29 (0.89, 1.91)	0.99 (0.72, 1.36)
3 or more	1.12 (0.59, 2.13)	1.32 (0.69, 2.51)	1.48 (0.92, 2.37)
**Birth order**			
First	Ref	Ref	Ref
Second	1.33 (0.97, 1.84)	0.26 (0.19, 0.37)***	0.66 (0.51, 0.85)**
Third or other	1.99 (1.21, 3.27)**	0.26 (0.14, 0.49)***	1.29 (0.86, 1.92)
**Family location**			
Urban	Ref	Ref	Ref
Rural	0.99 (0.73, 1.35)	1.01 (0.73, 1.38)	1.31 (0.99, 1.71)
**Parental education**			
Primary school or less	Ref	Ref	Ref
Junior high school	0.79 (0.55, 1.14)	1.19 (0.76, 1.85)	1.01 (0.73, 1.40)
Senior high school	0.91 (0.59, 1.39)	1.37 (0.84, 2.25)	0.77 (0.52, 1.15)
College or more	1.06 (0.52, 2.14)	1.23 (0.60, 2.51)	0.63 (0.31, 1.27)
**Perceived family income**			
Poor	Ref	Ref	Ref
Medium	0.64 (0.45, 0.89)*	1.09 (0.72, 1.63)	1.01 (0.74, 1.38)
Good	0.82 (0.46, 1.45)	1.74 (0.99, 3.07)	0.91 (0.53, 1.56)
**Parental violence**			
No	Ref	Ref	Ref
Yes	2.27 (1.54, 3.34)***	1.70 (1.10, 2.63)*	1.67 (1.16, 2.41)**
**Parent–child violence**			
No	Ref	Ref	Ref
Yes	2.08 (1.21, 3.58)**	2.82 (1.65, 4.83)***	5.03 (3.36, 7.51)***
**Son preference**			
No	Ref	Ref	Ref
Yes	1.90 (1.41, 2.58)***	1.27 (0.91, 1.78)	2.29 (1.79, 2.92)***

*p < 0.05, **p < 0.01, ***p < 0.001. Ref, Reference.

For girls, those living with a single parent were at greater risk of being a victim (OR = 1.81, 95% CI 1.06–3.11) than those living with two parents. Those who were left behind by both father and mother had increased odds of being a victim (OR = 1.57, 95% CI 1.12–2.21). What’s more, having a male sibling was significantly associated with being a victim (OR = 1.37, 95% CI 1.03–1.83). Age difference between siblings was a protective factor of being a bully-victim. Children born third or more were at greater risk of being a victim than those first-born (OR = 2.00, 95% CI 1.13–3.55). Those who live in rural areas were at greater risk of being victims than those who live in urban (OR = 1.33, 95% CI 1.04–1.70). Parental violence was risk factor of being a victim (OR = 1.68, 95% CI 1.16–2.43). Moreover, experiencing parent–child violence and son preference were risk factors of being a victim, a bully and a bully-victim of sibling bullying (Table 4).

**TABLE 4:** Multinomial logistic regression of roles of sibling bullying among girls (*N* = 3456).

Factors	Pure victim	Pure bully	Bully-victim
**Family composition**			
Two parents	Ref	Ref	Ref
Single parent	1.81 (1.06, 3.11)*	1.14 (0.59, 2.20)	1.05 (0.62, 1.78)
Adopted or other	0.59 (0.30, 1.17)	1.16 (0.72, 1.87)	0.99 (0.64, 1.54)
**Parental absence**			
No	Ref	Ref	Ref
Father	1.06 (0.72, 1.58)	0.88 (0.60, 1.29)	1.04 (0.76, 1.42)
Mother	1.21 (0.64, 2.29)	1.21 (0.65, 2.25)	1.39 (0.85, 2.26)
Both	1.57 (1.12, 2.21)**	1.06 (0.76, 1.48)	1.27 (0.97, 1.66)
**Sibling’s gender**			
Girl	Ref	Ref	Ref
Boy	1.37 (1.03, 1.83)*	1.30 (0.99, 1.70)	1.24 (0.99, 1.55)
**Age difference**			
0–4	Ref	Ref	Ref
5 or more	0.82 (0.61, 1.09)	0.99 (0.75, 1.31)	0.47 (0.38, 0.59)***
**Number of siblings**			
1	Ref	Ref	Ref
2	1.18 (0.85, 1.63)	1.74 (1.29, 2.33)***	1.25 (0.97, 1.60)
3 or more	1.04 (0.58, 1.90)	1.54 (0.90, 2.64)	1.51 (1.00, 2.26)
**Birth order**			
First	Ref	Ref	Ref
Second	1.13 (0.84, 1.50)	0.31 (0.23, 0.42)***	0.62 (0.49, 0.79)***
Third or other	2.00 (1.13, 3.55)*	0.37 (0.19, 0.71)**	1.05 (0.67, 1.65)
**Family location**			
Urban	Ref	Ref	Ref
Rural	1.19 (0.88, 1.61)	1.06 (0.80, 1.41)	1.33 (1.04, 1.70)*
**Parental education**			
Primary school or less	Ref	Ref	Ref
Junior high school	1.05 (0.73, 1.51)	0.95 (0.66, 1.36)	1.22 (0.90, 1.65)
Senior high school	1.00 (0.64, 1.58)	1.09 (0.72, 1.66)	1.20 (0.83, 0.73)
College or more	1.35 (0.66, 2.73)	1.19 (0.64, 2.22)	1.37 (0.76, 2.49)
**Perceived family income**			
Poor	Ref	Ref	Ref
Medium	0.73 (0.51, 1.03)	1.01 (0.70, 1.46)	0.85 (0.64, 1.13)
Good	0.61 (0.31, 1.20)	1.51 (0.88, 2.61)	0.89 (0.54, 1.48)
**Parental violence**			
No	Ref	Ref	Ref
Yes	1.68 (1.16, 2.43)**	1.01 (0.68, 1.51)	1.29 (0.93, 1.79)
**Parent–child violence**			
No	Ref	Ref	Ref
Yes	2.76 (1.15, 5.28)**	2.28 (1.10, 4.70)*	3.41 (2.00, 5.81)***
**Son preference**			
No	Ref	Ref	Ref
Yes	2.17 (1.66, 2.84)***	1.86 (1.44, 2.40)***	2.54 (2.05, 3.14)***

*p < 0.05, **p < 0.01, ***p < 0.001. Ref, Reference.

## Discussion

This is the first study to investigate the prevalence of sibling bullying based on the sample of Chinese children and adolescents. Over a quarter of children and adolescents were involved in sibling bullying in the last 6 mouths, and most of them were bully-victims. The finding is consistent with the previous studies which suggested that being both a victim and perpetrator was the most frequent role of sibling bullying involvement (Wolke and Skew, 2012; Jasmin and Anat, 2018). The possible explanations could include: first, the change of roles in sibling bullying involvement might owing to a fluid power dynamic that siblings usually gain more resource than each other by their familiarity (Wolke et al., 2015). Moreover, due to it is hard for victims to escape from sibling bullying behavior, conversely, they may act aggressive behavior against their siblings to protect themselves in the way learning from sibling’s perpetration.

This study found that boys were at greater risk of being pure victims in sibling bullying. There were no gender differences in pure bullies and bullying-victims. This is inconsistent with previous studies that boys were more likely to be bullies in the sibling bullying (Ersilia et al., 2011; Toseeb et al., 2020). This may be related to the different cultures. In the Chinese cultural context of collectivism and son preference, boys are more likely to be overprotected or coddled by their parent, which may inhibit boys’ aggression at home. This requires further cross-cultural research.

According to the combination of family systems theory and social learning theory, family environment plays an important role in children’s growth and development (Dantchev et al., 2018). In the study, family composition is a predictor of sibling bullying.

Consistent with previous research that age differences and birth order had the same effect on boys and girls (Tucker et al., 2013). But we were surprised to find that there are a lot of gender differences in the influence of family structure on sibling bullying.

First of all, those children living with a single parent were at greater odds of being pure bullies for boys, and pure victims for girls. The finding is in contrast to the prior work, which reveal that living in a single-parent family or a stepfamily has no significant association with aggressive behavior between siblings (Wolke et al., 2015). The inconsistency could be interpreted from different family composition of participants between the West and China. Tucker et al. (2014b) found that over one fifth of children live with a single parent in western countries, while the percent is far more than that in China from the present study (4.2%).

The second, parental absence is a risk factor of sibling bullying, and being left behind by both father and mother is related to being pure bullies and bully-victims for boys and pure victims for girls. Existing studies have found that long-term parental absence is associated with poor well-being of children because of inadequate family bonding, emotional vulnerability, and exposure to violence (Givaudan and Pick, 2013; Amato and Anthony, 2014), and left-behind children are at greater risk of psychological abuse and neglect, mental health problems, and behavioral problems (Eriksen and Jensen, 2009; Button and Gealt, 2010). The finding of this study extends our understanding of adverse status of left-behind children. Therefore, it may be practical to reduce sibling bullying among left-behind children by giving sufficient care and supervision from their parents.

The third, children whose sibling was a brother had higher odds of being all three roles of sibling bullying involvement for boys, and pure victims for girls. Consistent with the previous studies that brother–brother sibling were more likely to experience multiple incidents (Tucker et al., 2013), and girls with a male siblings are at higher risk of being victims (Menesini et al., 2011). These sex differences may show that boys are more aggressive, competitive, and sensitive to each other’s status, studies have shown more tensions between males (Zhang, 2020). While girls are raised to be easy-going and may be seen as a easy target of bullying in the family (Liu et al., 2021).

In our study, overall economic factors play a relatively small role in the impact of sibling bullying, but children living in economically disadvantaged families may indeed experience a greater risk of sibling bullying. Boys from poor-income families were at higher risk of being victims than those from medium-income families. Girls living in rural areas were at greater odds of being bully-victims than those living in urban. This is consistent with previous research that children from low-income families were more involved in sibling bullying (Bowes et al., 2014). Parents of low-income families were busy making a living and pay less attention to their children (Kochanova et al., 2022), which increases the likelihood of children being bullied by others or sibling due to the lack of parental supervision. Especially for girls in rural China, who have been taught from an early age to take responsibility for their families and be humble to their fellow family members, they are more likely to become victims of sibling bullying. Of course, they may also bully their sibling for limited parental resources.

Within three aspects of family environment, parental behavior have the most robust link to sibling bullying. Consistent with prior findings (Tucker et al., 2014a; Tippett and Wolke, 2015), both parent–parent violence and parent–child violence can predict greater risk of sibling bullying involvement, though girls were only at higher odds of being a pure victim compared with those girls who reported neither victimization nor perpetration. Conflict and aggression between parents as well as between a parent and a child could build a insecure climate within household (Zeng and Hesketh, 2016), which may lead to poor sibling relationship and even aggression between siblings (Piotrowski et al., 2021). This finding has a important implication for intervention of sibling bullying, which is that anti-bullying programs at home should not only help to improve sibling relationship but also take account of building harmonious parent–parent and parent–child relationship at the same time.

Son preference is a risk factor of sibling bullying. Surprisingly, those boys whose parents overtly value sons more than daughters were at greater risk of being a pure victim but were not more likely to be a pure bully compared with those boys whose parents had no behavior of son preference. What’s more, for girls, son preference increased odds of being all roles of sibling bullying involvement. The interesting finding may comes from two aspects. First, parental son preference would break the balance of sibling relationship and increase unequal interaction between boys and girls within household. Second, different parental treatment for sons and daughters may motivate those girls to perpetrate bullying behavior toward their brothers for obtaining sufficient resources and improving her status at home. Parents’ preference for sons is common in countries in East Asia through South Asia, to the Middle East and North Africa. In China, son preference stems from deep-rooted Confucian values and patriarchal family systems (Das Gupta et al., 2003), which could lead to discrimination against girls and neglect of health care and nutrition (Guo et al., 2015). Besides, the present study provides a new insight into negative influence of son preference, and indicates the significant association between parental son preference and sibling bullying of children.

Despite the potential contributions to the knowledge of sibling bullying, there were several limitations of the study. First, the cross-sectional nature limits our study to draw causality between sibling bullying and risk family characteristics. Second, this is a exploratory study to examine the association between sibling bullying and son preference, and we have not comprehensively control the potential effect of sibling dyad. Future studies should exclude brother–brother and sister–sister sibling dyad and further explode whether there is gender difference in the association among the children who have only one opposite-sex sibling. Finally, the study was conducted in a limited geographical setting. The extent to which this sample represents is also unclear, because information of respondents was only collected from children and adolescents in Hunan Province, central China. Future studies can further increase the sample size and recruit a national representative sample from different regions of China.

## Conclusion

In this study, we first explored family factors of sibling bullying in the context of eastern tradition and culture by using a large sample of Chinese children and adolescents. Our findings indicate that a number of people have experienced sibling bullying perpetration, sibling bullying victimization in this adolescent sample. Boys are at greater risk of becoming victims. The prevalence of sibling bullying indicates that bullying behavior between siblings has become an important public health issue with the implement of the two-child policy in China. Most important, the findings contribute new information about the association between sibling bullying and family environment, and we explore sex differences in sibling bullying in the context of Chinese culture. Furthermore, the study has practical implications for intervention of sibling bullying in China. Specifically, preventive efforts should be aimed at those children who live in the family with a single parent, parental absence of both father and mother, a male sibling, a sibling closing in age, parent–parent violence, parent–children violence, and son preference. This highlights the need for parents and health professionals to educate these children to learn to control behavior, and establish healthy and positive sibling relationships. At the same time, it is necessary to encourage their parents to eliminate son preference and construct a harmonious and positive family environment.

## Data Availability Statement

The original contributions presented in this study are included in the article/supplementary material, further inquiries can be directed to the corresponding author.

## Ethics Statement

The studies involving human participants were reviewed and approved by the Ethical Committee of Xiangya School of Public Health, Central South University. Written informed consent to participate in the study was provided by the participants’ legal guardian/next of kin.

## Author Contributions

ZQ and XL: study the conception and design. ZQ: data collection and draft manuscript preparation. YM: analysis and interpretation of results. All authors reviewed the results and approved the final version of the manuscript.

## Conflict of Interest

The authors declare that the research was conducted in the absence of any commercial or financial relationships that could be construed as a potential conflict of interest.

## Publisher’s Note

All claims expressed in this article are solely those of the authors and do not necessarily represent those of their affiliated organizations, or those of the publisher, the editors and the reviewers. Any product that may be evaluated in this article, or claim that may be made by its manufacturer, is not guaranteed or endorsed by the publisher.
